# Automated Microdosing System for Integration With a Miniaturized High-pressure Reactor System

**DOI:** 10.1155/JAMMC.2005.230

**Published:** 2005

**Authors:** Norbert Stoll, Ihsan Hawali, Kerstin Thurow

**Affiliations:** 1 Institute of Automation University of Rostock Richard Wagner Strasse 31 Rostock 18119 Germany; 2 Center for Life Science Automation (CELISCA) Friedrich-Barnewitz-Strasse 8 Rostock 18119 Germany

## Abstract

We present a new automated dosing system developed by the Institute for Automation of the University of Rostock, Germany. The new system is designed for the dosing of chemical liquids in the range of 50 μL–2.5 mL. It is integrated into a miniaturized reactor system to be used in the field of combinatorial synthesis. The reactor system can be pressurized up to 150 bar and tempered up to 200^^^C. A wide range of liquids with different physical properties can be handled with the new dosing system. A detailed description of the new dosing system in terms of function and operation as well as the relevant features and potential benefits is provided.


Journal of Automated Methods & Management in Chemistry, **2005** (2005), no. 4, 230–234
© 2005 Hindawi Publishing Corporation

### Automated Microdosing System for Integration With a Miniaturized High-pressure Reactor System

Norbert Stoll,[1] Ihsan Hawali,[1] and Kerstin Thurow[2]

[1] *Institute of Automation, University of Rostock, Richard Wagner Strasse 31, 18119 Rostock, Germany*
[2] *Center for Life Science Automation (CELISCA), Friedrich-Barnewitz-Strasse 8, 18119 Rostock, Germany*

*Received 6 June 2005; Accepted 13 June 2005*

We present a new automated dosing system developed by the Institute for Automation of the University of Rostock, Germany. The new system is designed for the dosing of chemical liquids in the range of 50 $\mu$L–2.5 mL. It is integrated into a miniaturized reactor system to be used in the field of combinatorial synthesis. The reactor system can be pressurized up to 150 bar and tempered up to 200°C. A wide range of liquids with different physical properties can be handled with the new dosing system. A detailed description of the new dosing system in terms of function and operation as well as the relevant features and potential benefits is provided.

### 1. INTRODUCTION

Materials research, biology, and chemical industry play the most important role in our modern life. In the recent times, a number of new technologies have been developed to aid modern laboratory research. High throughput screening (HTS) and combinatorial chemistry are good examples. Combinatorial chemistry is generally used to discover, for example, new reactions and catalysts because it facilitates the simultaneous generation and testing of a large number of compounds. However, it is quite challenging to establish new techniques for rapid and reliable dosing of different fluids in the reactor containers. These techniques have to meet combinatorial reaction requirements, in special conditions, that is, in combinatorial catalysis such as high reaction pressure and temperature, dealing with low volumes, and the reliable and accurate dosing of different kinds of chemical reagents.

### 1.1. *Liquid-handling systems*

In the modern laboratory it is very important to be able to reduce the material volumes. Beside cost reduction due to consumption of expensive reagents, there are other crucial reasons to reduce the material volumes. These include reduction in the waste disposal costs, reduction in the time of screening, and reduction in the well-plate storage costs. Therefore, it is vital to control the handling of lower liquid volumes as well as reducing these volumes as much as possible with high reliability, high accuracy, low service cost, and highly parallel fashion.

Recently, various liquid-handling systems have been developed to solve the problem of dosing low volumes of different kinds of fluids. Some of them are quite similar to ink-jet print heads so they use drop-on-demand technology for injecting single liquid drops in the range of hundreds of picoliters. Several interesting low-volume liquid-handling technologies are based on solenoid valves sometimes in combination with other drivers. The limitation of these technologies regarding the combinatorial reaction requirements lies in their use under atmospheric pressure.

Conventional pumps like microrotary pumps found in the market could dose high-viscose liquids only up to 40 bar. Another example are syringe pumps, they are capable of dosing reliably under high pressure for a wide range of different liquids, but they are not suitable to be integrated with our miniaturized reaction system. Additionally, problems will appear in order to keep the sealing of the reaction system from the outside environment. Therefore, there is a need to develop a new microsystem that fulfils the combinatorial reaction requirements listed previously.

### 1.2. *Requirements of the new microdosing system*

The miniaturized high-pressure reactor system consists of 8 reactors with 2.5 mL internal volumes each. It is used in combinatorial chemistry to discover new reactions and catalysts. With the help of an automatic control, this system can be pressurized up to 150 bar and heated up to 200°C. Figure 1 shows the principle of the miniaturized high-pressure reactor system.

Correspondence and reprint requests to Norbert Stoll, Institute of Automation, University of Rostock, Richard Wagner Strasse 31, 18119 Rostock, Germany; E-mail: norbert.stoll@uni-rostock.de.

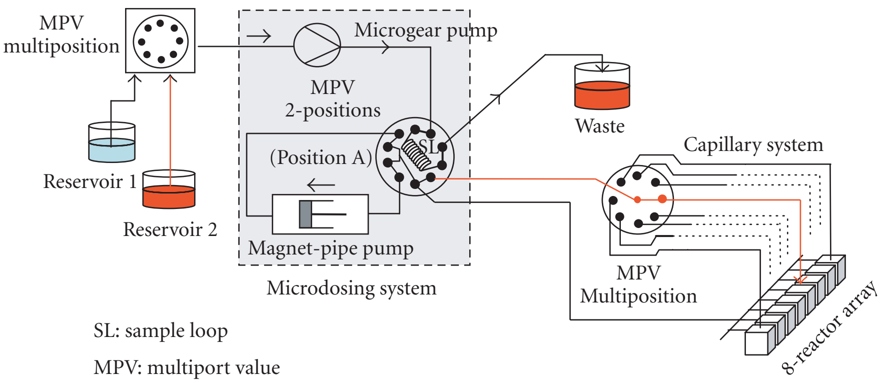

Figure 1: Integration of MDS with the reactor system.

The new liquid-handling system had to be developed to dose a wide range of compounds used in combinatorial chemistry in the reactor containers. The dosing process has to be performed automatically either before or after building up high pressure in the reaction system. The new MDS is terminated to a controller, in our case a personal computer. Moreover, the new microdosing system must meet the above listed requirements of liquid-handling systems.

### 2. OPERATING PRINCIPLE

With the development of the new MDS, two important problems arise. First, the precise handling of liquids in the microliter range, and the second is the ability to perform liquid dosing when the reactor system is set under high pressure.

Figure 2 shows the schematic diagram of the operating principle of the new microdosing system. It consists of three main components

(i) A conventional micropump, which is a microgear pump.
(ii) A two-position multiport valve.
(iii) A self-designed magnet-pipe pump based on the syringe technology.

Additionally a sample loop is used to store the amount of liquid that needs to be injected into the reactor's container. The liquid volumes that are injected into the reactor container match the volume of the sample loop used. A stainless steel capillary is used to connect all the microdosing system components to each other. It has an internal diameter of 0.88 mm.

At the core of the MDS is a magnet-pipe pump that is based on syringe technology. As shown in Figure 2, the magnet-pipe pump has a 10 mm diameter piston that moves in a 10 cm long stainless steel pipe. The piston is made of magnet material coated with stainless steel hollow case. A linear-moving system moves an outside magnet ring around the pipe. The piston can move inside the pipe body as the linear system moves the outside magnet ring. The movement of the piston from its start position to its end position is used to inject the liquid inside the sample loop into the reactor container.

The multiport valve separates the reaction system from the outside environment. The valve is cased in a way that the system components connected to it are arranged in two independent separated loops in order to seal the reaction environment from outside one. The first loop (Loop 1) is an atmosphere loop when the valve is switched to position A. In this loop the sample loop is connected to the micro gear pump and the waste. In position B, the loop 2 (the high-pressure loop) is loaded. In this loop the sample loop is connected to the reactor and to the magnet-pipe pump. Figures 3 and 4 show a schematic drawing of these two loops. The atmosphere path includes liquid reservoir, microgear pump, and the sample loop.

### 2.1. *The dosing process*

The way of dosing the liquid using the microdosing system is based on consecutive steps performed by the system components, as shown in Figures 2 and 3.

First of all, the multiports valve is in the position A. In this position the sample loop is connected to the microgear pump and the waste. The pump transfers the liquid from its reservoir into the sample loop for a short time (Figure 2). Secondly, by the actuation of the multiport valve, the latter switches its position into position B. In this case, the sample loop connects to the high-pressure loop. By actuating the magnet pump, a piston transfers the liquid from the sample loop direct into the reactor container (Figure 3).

### 2.2. *Experiments and measurements*

Different sample loop volumes (100, 250, and 500 $\mu$L) were used to test the microdosing system under atmospheric pressure as well as under high pressure up to 100 bar. One sample loop volume is used to vary the dosed volume via one dosing event. Liquid properties like viscosity and density essentially influence the flow resistances of the system capillary. Thus, different liquids are used to test the effect of liquid properties on the microdosing system. These liquids are water, DMSO, and 1-octanol. Additionally, various measurements

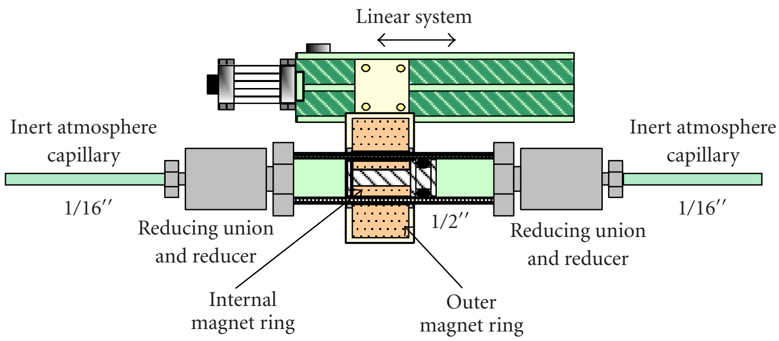

Figure 2: Schematic operating principle for the magnet-pipe pump.

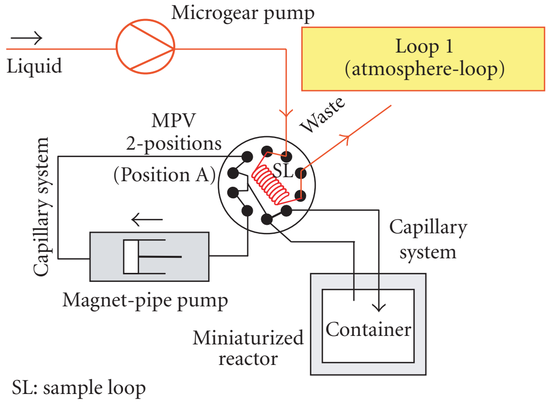

Figure 3: Schematic drawing of the atmosphere-loop when the multiports valves are in position A.

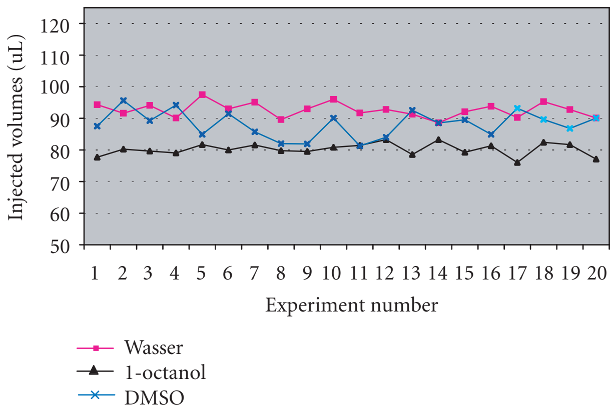

Figure 5: Reproducibility by using a 100 $\mu$L sample loop.

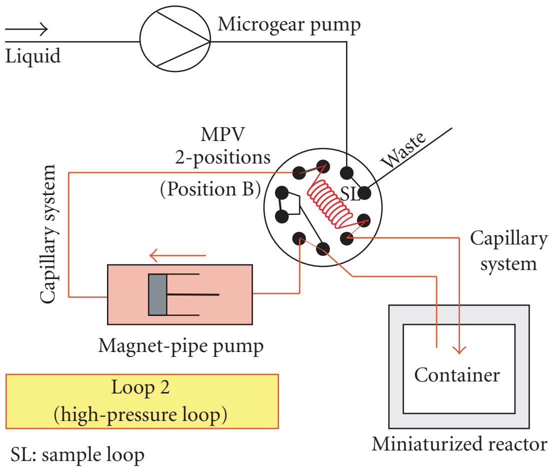

Figure 4: Schematic drawing of the atmosphere-loop when the multiports valves are in position B.

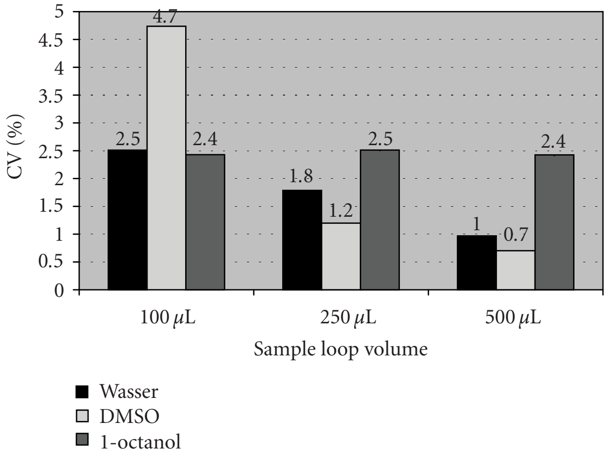

Figure 6: Injection precision (CV) by using different sample loops under atmospheric pressure.

were carried out with the dosing system for characterizing and proving its reliability.

The reproducibility is usually characterized by the parameters of the dispensed volumes after various dispensing events. Results of the reproducibility measurements under atmospheric pressure are shown in Figures 5 and 6. Figure 5 gives an overview of the results of successive dosing events for sample loop with 100 $\mu$L volume using different liquids. Figure 6 shows low CV% values between 0.7% and 5% from dosing average values for all tested liquids and sample loops. Generally, using sample loops with larger volumes

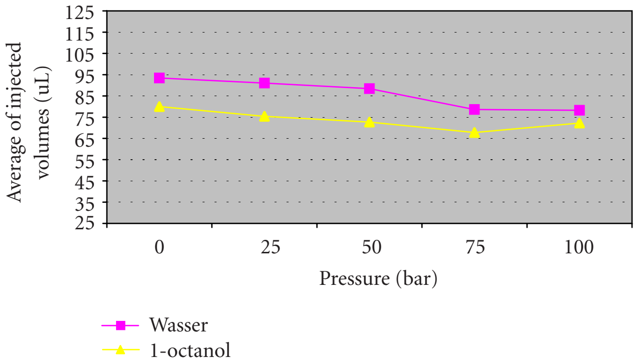

FIGURE 7: Average of the injected volumes by using a 100 $\mu$L sample loop under high pressure.

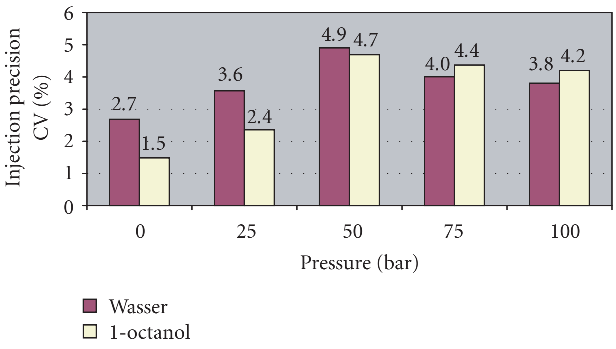

FIGURE 8: Injection precision (CV) by using 100 $\mu$L sample loops for all tested high-pressure values.

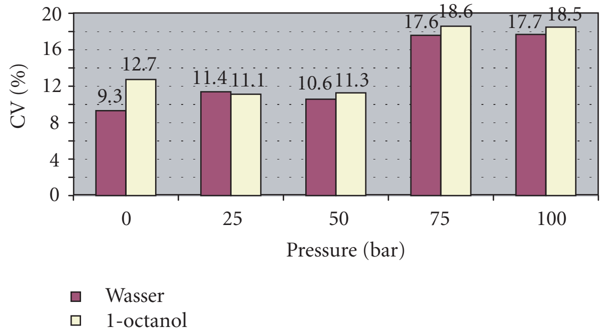

FIGURE 9: Injection precision (CV) by using 25 $\mu$L sample loop for all tested high-pressure values.

gives better reproducibility than using sample loops with smaller volumes.

Secondly, the influence of the high pressure on the dosing performance has been proven experimentally. As mentioned above different sample loops, liquids, and high-pressure conditions are used to test the microdosing system. The pressure value varies from atmospheric pressure up to 100 bar in steps of 25 bar. Figure 7 shows the results of successive dosing events for a selected sample loop (100 $\mu$L). It shows that the dosing volumes decreased slowly with increasing pressure.

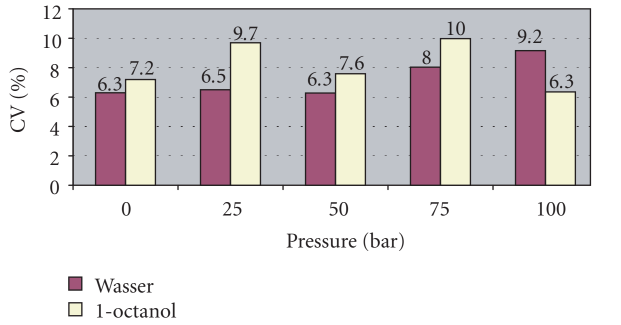

FIGURE 10: Injection precision (CV) by using 50 $\mu$L sample loop for all tested high-pressure values.

The average value varies with the viscosity of the liquids. The higher the viscosity of the liquid, the higher the average value that can be dosed for the same sample loop. Figure 8 compares the CV of dosing volumes for same dosing conditions. It shows a slight increase in a CV with high pressure. The CV increases from 2.7%–4.9% with water and from 1.5%–4.7% with 1-octanol that has a higher viscosity.

In order to define the minimal volumes that can be handled using the microdosing system, sample loops of 25 and 50 $\mu$L volumes were used (Figure 9). These experiments were done under either atmospheric or high pressure. The results obtained using a sample loop of 25 $\mu$L show significant high errors. Higher values of the working high pressure gives lower dosing precision. The dosing precision is under 12% of sample loop volume under high pressure up to 50 bar then it increases up to 19% by 100 bar. However, by using the sample loop of 50 $\mu$L the results show that the microdosing system can be used to handle a liquid volume up to 50 $\mu$L with an acceptable dosing precision of $< 10\%$ (Figure 10).

### 3. CONCLUSION

A new microdosing method for liquid handling under high-pressure environment has been presented in detail. An experimental evidence for its performance has been given too. Different liquids have been dosed in the miniaturized reaction container. The container can be pressurized up to 100 bar. The precision of the dosing volumes was shown to be within 5% of the different sample loop volumes and high pressure values that are used in these experiments. The dosing precisions depend slightly on the high pressure and on the viscosity of the liquids.

### REFERENCES

[1] M. Mandlehr, "Parallele Prozessentwicklung," *LaborPraxis*, vol. 2002, no. 10, pp. 82–84, 2002.

[2] R. Lemke, "Automation eines miniaturisierten Hochdruckreaktionssystems," Ph.D. dissertation, Fakultät für Ingenieurswissenschaften der Universität Rostock, Rostock, Germany, 2002.

[3] P. Gravesen, J. Branebjerg, and O. S. Jensen, "Microfluidics-a review," *Journal of Micromechanics and Microengineering*, vol. 3, no. 4, pp. 168–182, 1993.

[4] Technical Note 99-01: "Background on Ink-Jet Technology", MicroFab Technologies, Plano, Tex, USA, 1999.
[5] G. Vetter, *Handbuch Dosieren*, Vulkan, Essen, Germany, 2nd edition, 2003.
[6] T. C. Dickenson, Ed., *Pumping Manual*, Elsevier Science, Oxford, UK, 9th edition, 1995.
[7] Gesellschaft für Mikrodosiersysteme mbH, "Mikrodosiererung mit Piezo- und Ventil-Technologie", http://www.microdrop.de/html/technologien.html.

